# Inflammatory peritonitis secondary to rupture of borderline mucinous ovarian tumor

**DOI:** 10.1259/bjrcr.20180085

**Published:** 2018-11-14

**Authors:** Rebeca Sigüenza González, María Pina Pallín, Teresa Álvarez De Eulate García

**Affiliations:** 1 Department of Radiology, Clinical Hospital, Valladolid, Spain

## Abstract

We present the case of a woman with a history of untreated borderline ovarian tumor. She went to the emergency department with abdominal pain and vomiting. In this context, the first diagnostic possibilities to rule out were tumor progression and/or tumor complication. Inflammatory peritonitis secondary to a ruptured ovarian tumor is a complication that has not been widely discussed. It is a surgical emergency. The differential diagnosis should be made with peritoneal carcinomatosis. The main radiological finding that should make us to suspect this entity is the reduction of tumor’s size in an untreated ovarian mass.

## Clinical presentation

**Figure 1.**
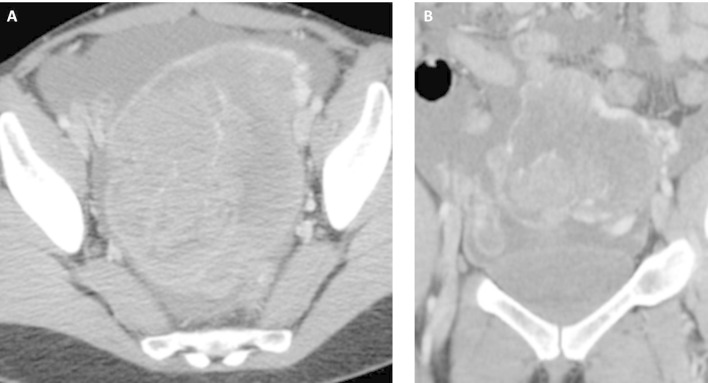
Axial (A) and coronal (B) scans of abdominal CT which shows ovarian tumor at first time. It is a big and heterogeneous mass located in lower pelvis. It is a cystic mass with solid component in its upper margin.

**Figure 2.**
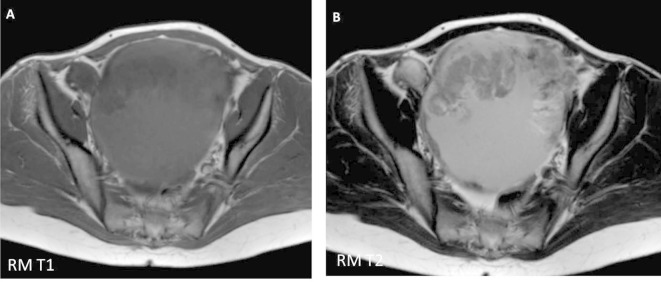
Axial scans of abdominal RM with T1 (A) and T2 (B) weight sequences. It confirms the existence of a cystic mass (hypointense in T1 and hyperintense in T2) with solid component in its upper margin (hypointense in T1 and T2).

Female, 38 years old, with mucinous borderline ovarian tumor, which had not yet been treated. Her clinical history included diagnostic CT and MRI and CT for tumor staging (Figures 1 and 2). Some weeks after tumor was diagnosed, she presented at emergency department with abdominal pain, fever and vomiting. Blood tests showed an increasing of leukocytes and C-reactive protein.

## Differential diagnostic

In relation to the clinical context presented, the initial differential diagnosis included progression of ovarian neoplasm (peritoneal carcinomatosis) and tumor complication.

## Imaging findings

**Figure 3.**
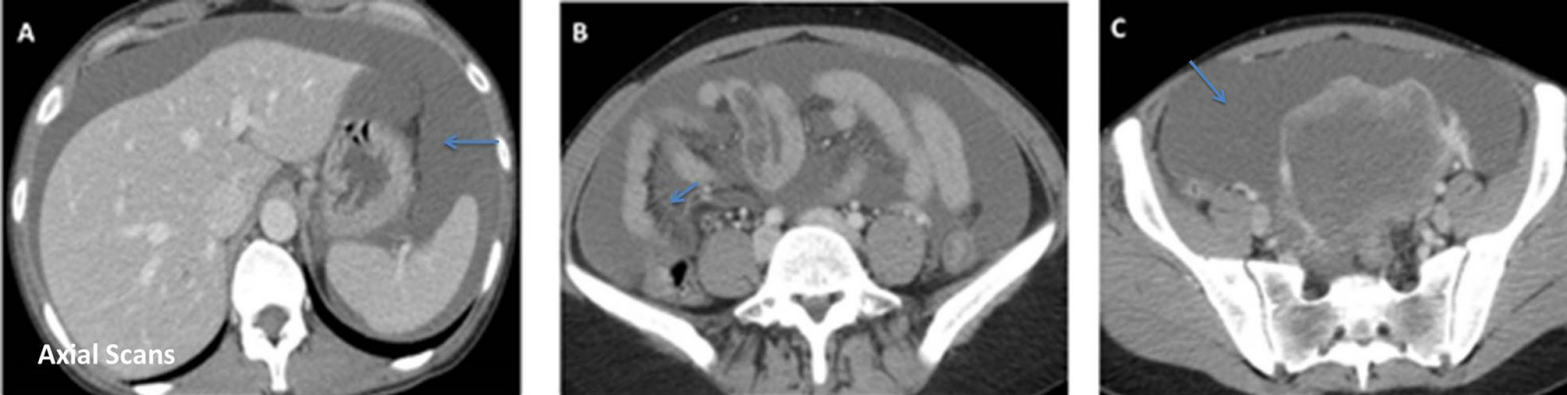
Axial scans of emergency abdominal CT, which shows abundant ascitic liquid (arrows in A and C) and hyperdensity of abdominal fat (arrows inB).

**Figure 4.**
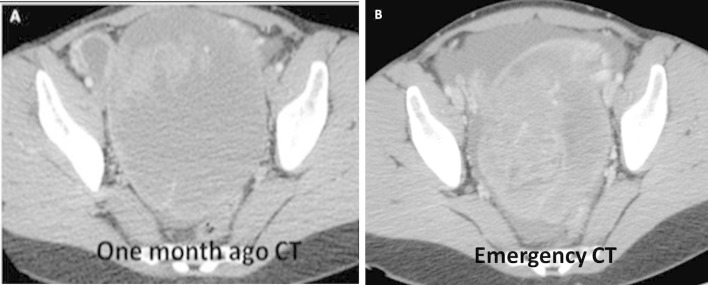
Axial scans which compare staging (A) and emergency CT (B). In emergency CT a reduction in tumor’s size was observed (B).

An emergency abdominal CT with intravenous contrast was performed. It showed ascitic liquid (arrows in Figure 3A and C), peritoneal enhancement and hyperdensity of abdominal fat (arrows in Figure 3B). In addition, the comparison with previous CT (Figure 4A) performed 1 month ago showed a decreased of mass size (Figure 4B). For these reasons, the first possibility was inflammatory peritonitis secondary to ovarian tumor rupture.

## Treatment

The patient was undergone to surgery with left anexectomy and intestinal resection (since the lesion was attached to the sigmoid). Histology confirmed that it was a ruptured mucinous ovarian tumor with associated inflammatory peritonitis.

## Outcome, follow up, and discussion

Mucinous ovarian tumors account for 10–15% of all ovarian tumors.^1^ About 80% are considered benign, in the remaining 20%, borderline tumors and carcinomas are included. They are more frequent in elderly females. Histologically, they are composed of complex papillae lined by columnar epithelium with mucinous contents, similar to those present in the endocervical glands. Their nuclei show slight atypia. Its clinical manifestations are nonspecific (asthenia, adominal pain, menstrual alterations, abdominal distension).

From the radiological point of view, the mucinous tumor manifests as a multilocular cystic mass with thin, regular walls and septa. It can present a heterogeneous aspect and contain liquid with different signal intensities/densities but it will not present nodules or vegetations of intra/ extracystic growth.^2^ The average size of these tumors is variable and ranges from 2 to 36 cm, being often 8–10 cm at the time of diagnosis.

Borderline mucinous tumors show a low potential for malignancy with a higher proliferative index than mucinous cystadenomas but without stromal invasion. They are less frequent than the latter but more common than carcinomas.^3^

The complications of ovarian tumors, especially those of a benign nature or with a low degree of malignancy, have not been widely discussed in the literature. Regarding the case presented, in which the diagnosis of inflammatory peritonitis secondary to the rupture of a mucinous ovarian tumor was demonstrated, we intend to emphasize this complication. The acute supposes a surgical emergency. It is a rare complication, more frequently described in the context of mature cystic teratomas.^4^ It can be spontaneous (as in the case described) or occur in the context of iatrogenesis, infection, trauma or torsion. It is necessary to differentiate if it shows an acute or chronic character.

In our experience, the diagnostic key in the acute form is represented by abdominal pain with peritoneal irritation and an untreated tumor mass that has decreased in volume compared to previous studies. The isolated finding of abundant intraabdominal ascitic fluid isn’t diagnostic. It is common to find it in the context of uncomplicated ovarian tumors with associated peritoneal carcinomatosis. Therefore, the comparative assessment of tumor size with previous studies is key in the diagnosis of this disease, as long as the tumor mass has not been treated.

The chronic form is characterized by small “leaks” of intratumoral fluid of long evolution through weaknesses of the cystic wall of the lesion.^4^

The differential diagnosis should be carried out with peritoneal carcinomatosis.

## Learning points

1. The complications of ovarian tumors hadn’t been widely discussed in the literature.
2. Ovarian tumor’s rupture is a surgical emergency.
3. The main differential diagnosis should be carried out with peritoneal carcinomatosis.
4. The radiological key in the diagnosis of an ovarian tumor rupture is represented by abdominal pain, peritoneal irritation and an untreated tumor mass that has decreased in volume compared to previous studies.
5. The isolated finding of ascitic fluid isn’t diagnostic. It is common to find it in peritoneal carcinomatosis without other tumor’s complications.
